# Development and internal validation of a clinical prediction model using machine learning algorithms for 90 day and 2 year mortality in femoral neck fracture patients aged 65 years or above

**DOI:** 10.1007/s00068-022-01981-4

**Published:** 2022-05-29

**Authors:** Jacobien Hillina Froukje Oosterhoff, Angelique Berit Marte Corlijn Savelberg, Aditya Vishwas Karhade, Benjamin Yaël Gravesteijn, Job Nicolaas Doornberg, Joseph Hasbrouck Schwab, Marilyn Heng

**Affiliations:** 1 Department of Orthopaedic Surgery, Amsterdam Movement Sciences, Amsterdam University Medical Centers, University of Amsterdam, Meibergdreef 9, 1105AZ Amsterdam, The Netherlands; 2 Department of Orthopaedic Surgery, Massachusetts General Hospital, Harvard Medical School, Boston, MA USA; 3 Department of Public Health, Erasmus University Medical Center, Rotterdam, The Netherlands; 4 Department of Orthopaedic Surgery, University Medical Centre Groningen, University of Groningen, Groningen, The Netherlands; 5 Harvard Medical School Orthopedic Trauma Initiative, Massachusetts General Hospital, Boston, MA USA

**Keywords:** Hip fracture, Femoral neck fracture, Geriatric trauma, Prediction model, Mortality, Machine learning, Precision medicine

## Abstract

**Purpose:**

Preoperative prediction of mortality in femoral neck fracture patients aged 65 years or above may be valuable in the treatment decision-making. A preoperative clinical prediction model can aid surgeons and patients in the shared decision-making process, and optimize care for elderly femoral neck fracture patients. This study aimed to develop and internally validate a clinical prediction model using machine learning (ML) algorithms for 90 day and 2 year mortality in femoral neck fracture patients aged 65 years or above.

**Methods:**

A retrospective cohort study at two trauma level I centers and three (non-level I) community hospitals was conducted to identify patients undergoing surgical fixation for a femoral neck fracture. Five different ML algorithms were developed and internally validated and assessed by discrimination, calibration, Brier score and decision curve analysis.

**Results:**

In total, 2478 patients were included with 90 day and 2 year mortality rates of 9.1% (*n* = 225) and 23.5% (*n* = 582) respectively. The models included patient characteristics, comorbidities and laboratory values. The stochastic gradient boosting algorithm had the best performance for 90 day mortality prediction, with good discrimination (c-statistic = 0.74), calibration (intercept = − 0.05, slope = 1.11) and Brier score (0.078). The elastic-net penalized logistic regression algorithm had the best performance for 2 year mortality prediction, with good discrimination (c-statistic = 0.70), calibration (intercept = − 0.03, slope = 0.89) and Brier score (0.16). The models were incorporated into a freely available web-based application, including individual patient explanations for interpretation of the model to understand the reasoning how the model made a certain prediction: https://sorg-apps.shinyapps.io/hipfracturemortality/

**Conclusions:**

The clinical prediction models show promise in estimating mortality prediction in elderly femoral neck fracture patients. External and prospective validation of the models may improve surgeon ability when faced with the treatment decision-making.

**Level of evidence:**

Prognostic Level II.

**Supplementary Information:**

The online version contains supplementary material available at 10.1007/s00068-022-01981-4.

## Introduction

The number of hip fractures continues to rise, and are predicted to an incidence of 6.26 million cases each year worldwide in 2050 [1]. Numerous patient and injury characteristics are associated with a high mortality rate after hip fracture, with incidences ranging from 14 to 35% in the first year [2–4]. The treatment decision for femoral neck fractures has been a frequent topic of discussion in the orthopedic literature, where the optimal treatment decision-making and implant choice remain challenging [5, 6].

Predicting mortality may guide which patient may benefit from arthroplasty surgery (hemi- or total hip arthroplasty), internal fixation (e.g. a sliding hip screw or cancellous screws) or nonoperative management [7, 8]. In patients aged 65 years or above, the decision between arthroplasty and internal fixation remains under debate, and optimal treatment may be individualized depending on patients’ preferences and goals, informed by the risk and benefits of treatment options [5, 6]. Long-term functional outcomes may be better in healthy older patients undergoing arthroplasty compared to internal fixation, with lower reoperation rates [9, 10]. A recent study showed that a shared decision-making process including non-operative management for a proximal femoral fracture might be a viable option for frail institutionalized patients with limited life expectancy [8]. Identifying patient and injury characteristics associated with mortality may aid surgeon, patients and family in shared decision-making and optimize care in femoral neck fracture patients [11]. In other words, a decision support tool to predict shorter- and longer-term mortality would allow for risk stratification of patients aged 65 years or above with femoral neck fractures to guide treatment decision-making.

Thus, an accurate preoperative prediction model may be required to efficiently target patients benefiting from a specific intervention and facilitate true shared decision-making based on personalized risks and benefits. Many mortality prediction models have been described in the geriatric trauma [12, 13] and hip fracture population [14–16], but only few studies predict mortality in the hip fracture patient beyond the 30-day period with good model performance [14]. Most hip fracture registries have a follow-up period of maximum 1 year [17], the use of institutionally collected data creates the opportunity to develop prediction models with longer follow-up. Prior prospective randomized controlled trials chose 2-year as the endpoint to account for longer follow-up for management of the acute hip fracture patient [6, 18]. In addition, clinical decision support using machine learning (ML) algorithms has been employed in the hip fracture population (e.g. 30 day mortality [16] or 30 day delirium [19] prediction), and has also shown to be useful in helping to predict outcomes in other areas including orthopaedic surgery [1–4, 20–22].

Therefore, this study aimed to develop and internally validate a clinical prediction model using machine learning algorithms for 90 day and 2 year mortality in femoral neck fracture patients aged 65 years or above.

## Materials and methods

### Data source

This retrospective cohort study was approved and registered with the institutional review board (IRB) prior study start-up. A search in the Research Patient Data Registry (RPDR) was performed to identify patients older than 65 years of age who underwent operative treatment for a femoral neck fracture, OTA type 31-B (as classified by the Orthopaedic Trauma Association (OTA) [23]), who presented to our institutions between January 2001 and December 2017. RPDR is a clinical data registry that collects medical records from institutions within the Partners Healthcare System and may be queried after IRB approval. Our institutions accounted for two level I trauma centers and three community (non-level I trauma) hospitals. Patients were excluded if presented with a pathological fracture.

### Primary outcomes

The primary outcome was 90 day and 2 year mortality in patients sustaining a femoral neck fracture, OTA type 31-B. Mortality was assessed by cross-referencing the Social Security Death Index (a database of people whose deaths were reported to the Social Security Administration) and through manual chart review. The time endpoints of 90 day and 2 year mortality were chosen on the basis of prior studies [6, 18, 24].

### Baseline data

The following preoperative variables were collected: age, gender, race, ethnicity, marital status, veteran status, side of injury, displacement of the fracture, Charlson Comorbidity Index, presence of comorbidities [myocardial infarction, congestive heart failure, peripheral vascular disease, cerebrovascular accident, dementia, chronic obstructive pulmonary disease, rheumatic disease, peptic ulcer disease, liver disease, diabetes, hemi- and paraplegia, renal disease, cancer, coagulopathy, drug abuse, alcohol abuse, depression], preoperative medication use [immunosuppressants, anti-coagulants, steroids, bisphosphonates, angiotensin converting enzyme inhibitors, angiotensin receptor blockers, beta blockers, beta-2 agonists, opioids] and laboratory characteristics [calcium(mg/dL), creatinine(mg/dL), hemoglobin(g/dL), potassium(mEq/L), platelet count(10^3^/µL), prothrombin time(PT), International Normalized Ratio (INR), white blood cell count(10^3^/µL), absolute lymphocyte count(10^3^/µL), absolute neutrophil count(10^3^/µL), neutrophil/lymphocyte ratio, platelet/lymphocyte ratio]. We did not assess peri- or postoperative variables as candidate input variables emphasizing the development of a preoperative prediction model to aid treatment decision-making.

Multiple imputation with the missForest methodology was used to impute variables with less than 30% missing data [25].

### Variable selection

Variable selection was performed to identify and select those preoperative variables contributing most to our outcome variable, conducted by entering all relevant explanatory variables into random forest algorithms with recursive selection [26]. Given the rule of thumb for developing prediction models with a binary outcome (those with and without the outcome), we ensured at least 10 events for each predictor variable included in the model [27].

### Development and internal validation of the clinical prediction model

The following ML algorithms were chosen for modeling based on prior research [19, 22, 28, 29]: Stochastic Gradient Boosting (SGM), Random Forest (RF), Support Vector Machine(SVM), Neural Network (NN) and Elastic-Net Penalized Logistic Regression (PLR).

Internal validation was carried out by performing a stratified 80:20 split of the dataset to create a training set (*n* = 1983) and a test set (*n* = 495). Subsequently, the algorithms were trained on the training set with ten-fold cross-validation repeated 3 times. Cross-validation means dividing data into a selected number of groups, named folds. First, the data are divided into 10 equally sized folds. Then, the algorithms were trained on 9 of the 10 folds (90% of the training data) and tested on the remaining fold (10% of the training data). Consecutively, performance was evaluated in the test dataset.

### Model performance

Model performance was evaluated according to a proposed framework for evaluation of a clinical prediction model [30] that includes: discrimination with the c-statistic, calibration slope and intercept (in line with the method by Cox [31]) and the overall performance with the Brier score.

The c-statistic (area under the curve of a receiver operating characteristic curve) is a score ranging from 0.50 to 1.0 with 1.0 indicating the highest discrimination score and 0.50 indicating the lowest. The higher the discrimination score, the better the model’s ability to distinguish patients who got the outcome from those who did not [32].

A calibration plot plots the estimated versus the observed probabilities for the primary outcome. A perfect calibration plot has an intercept of 0 (< 0 reflects overestimation, > 0 reflects underestimating the probability of the outcome) and a slope of 1 (model is performing similarly in training and test sets) [30, 33]. In a small dataset, slope is often < 1 reflecting model overfitting; probabilities are too extreme (low probability too low, high probability too high) [32].

The null-model Brier score, which equals the probability of mortality in the dataset, was used to benchmark the algorithm’s Brier score. A Brier score lower than the null-model Brier score indicates superior performance of theprediction model to this null benchmark. Perfect prediction would have a Brier score of 0 and 1 the poorest prediction [30].

### Decision curve analysis

In addition, decision curve analysis was undertaken and visualized to investigate the net benefit (weighted average of true positives and false positives) of the conducted algorithms over the range of risk thresholds for clinical decision-making [34]. The net benefit is a weighted average of true positives and false positives, formula = sensitivity x prevalence – (1-specificity) x (1 – prevalence) x odds at the threshold probability). With threshold probability, we refer to the probability that an algorithm ranks a ‘positive’ outcome over a ‘negative’ outcome. In this study, a ‘positive outcome’ is someone at high risk of mortality in 90 days or 2 years. If the threshold is set at 0.5, than patients with a probability > 0.5 are classified as ‘positive’, and < 0.5 are classified as ‘negative’. If the threshold is set at 0.8, then patients with a probability > 0.8 are classified as ‘positive’, and < 0.8 are classified as ‘negative’. The decision curve of the model is compared to decision curves of treating everyone as being at risk for shorter- or longer-term mortality (depending on the endpoint), and treating no one as being at risk.

For 90 day mortality, risk thresholds in the range of 1:3 (risk of 25%) to 1:5 (risk of 17%) seemed clinically relevant [35]. This effectively means we accept 3 to 5 cases of underestimation (a predicted probability that is too low for surviving up to 90 days, which may result in choosing a less invasive treatment option) per case of overestimation (a predicted probability that is too high for surviving up to 90 days, which may result in choosing a more invasive treatment option).

For 2 year mortality, higher risk thresholds, in the range of 1:2 (risk of 33%) to 1:3 (risk of 25%), seemed clinically relevant [35]. Not performing arthroplasty surgery in patients surviving up to 2 year is worse than in patients surviving up to 90 days. Therefore, we accept fewer cases of underestimation of the mortality probability.

### Open-access web-application and individual patient explanation

The best-performing algorithms across the model performance metrics as described above, for each primary outcome (i.e. 90 day and 2 year mortality), were deployed as an open-access web application accessible on desktops, tablets and smartphones.

Individual patient-level explanations are incorporated in the web application for interpretation of the model to understand the reasoning how the model made a certain prediction. Local model explainability helps in understanding which features of the patient contributed most to the model’s prediction [36].

### Statistical analysis

Categorical variables will be described as absolute numbers with frequencies, and continuous variables as medians with interquartile ranges (IQR). The model performance metrics were calculated with 95% confidence interval (CI). Given the retrospective study design, post hoc power analyses were conducted to evaluate the sample size of the study with an alpha value of 0.05.

### Guidelines

The study set-up has been performed following the Transparent Reporting of Multivariable Prediction Models for Individual Prognosis or Diagnosis Guideline (TRIPOD Statement) (Supplemental Table 1) [37].

### Software

Data pre-processing and analysis were performed using R Version 4.1 (“R: A Language and Environment for Statistical Computing” The R Foundation, Vienna, Austria 2013) and R-studio Version 1.2.1335 (R-Studio, Boston, MA, USA). Hyperparameter tuning was performed as recommended in the R package vignettes.

## Results

### Participants

In total, 2478 patients were included in this study with 90 day and 2 year mortality rates of 9.1% (*n* = 225) and 23.5% (*n* = 582) respectively. Of the included patients, 69.5% (*n* = 1723) patients were female, and the median age was 83 years (interquartile range = 76–88) (Table 1). The post hoc power analyses revealed 100% power in both evaluations (*α* = 0.05).

**Table 1:** Baseline characteristics of study population, *n* = 2478

Variable	*n* (%) | median (IQR)
Age	83 (76–88)
Female gender	1723 (69.5)
Race, white	2202 (94.3)
Ethnicity, hispanic	33 (1.4)
Marital status, married	931 (39.1)
Veteran	324 (16.1)
Side of injury, left	1273 (51.4)
Displaced fracture (Garden III–IV)	1765 (71.2)
Charlson comorbidity index	2 (0–3)
Comorbidities	
Myocardial infarction	379 (15.3)
Congestive heart failure	718 (29.0)
Peripheral vascular disease	417 (16.8)
Cerebrovascular accident	442 (17.8)
Dementia	309 (12.5)
Chronic obstructive pulmonary disease	658 (26.6)
Rheumatic disease	180 (7.3)
Peptic ulcer disease	57 (2.3)
Liver disease	129 (5.2)
Diabetes	477 (19.2)
Hemi paraplegia	60 (2.4)
Renal disease	494 (19.9)
Cancer	412 (16.6)
Coagulopathy	164 (6.6)
Drug abuse	69 (2.8)
Alcohol abuse	91 (3.7)
Depression	449 (18.1)
Medication	
Immunosuppressants	462 (18.6)
Anti-coagulants	1320 (53.3)
Steroids	409 (16.5)
Bisphosphonates	168 (6.8)
ACE inhibitors	602 (24.3)
Angiotensin receptor blockers	194 (7.8)
Beta blockers	1287 (51.9)
Beta-2 agonists	470 (19.0)
Opioids	1700 (68.6)
Laboratory characteristics	
Calcium	9.0 (8.6–9.4)
Creatinine	0.93 (0.74–1.21)
Hemoglobin	12.1 (11.0–17.8)
Potassium	4.0 (3.7–4.3)
Platelet	211 (168–269)
PT	35 (26–47)
INR	1.1 (1.0–1.2)
White blood cell count	9.6 (7.5–12.1)
Absolute lymphocyte	1.14 (0.82–1.55)
Absolute neutrophil	7.77 (5.62–8.27)
Neutrophil/lymphocyte ratio	6.7 (4.2–10.8)
Platelet/lymphocyte ratio	188.8 (132.8–261.4)
Mortality	
90 day	225 (9.1)
2 year	582 (23.5)

*n* number; *IQR* interquartile range

Rates of missing data for covariates were as follows: race (144, 5.8%), ethnicity (144, 5.8%), marital status (98, 4.0%), veteran status (465, 18.8%), calcium (394, 15.9%), creatinine (193, 7.8%), hemoglobin (194, 7.8%), potassium (200, 8.1%), platelet (196, 7.9%), PT (274, 11.1%), INR (386, 15.6%), white blood cell count (193, 7.8%), absolute lymphocyte (567, 22.9%), absolute neutrophil (491, 19.8%), neutrophil/lymphocyte ratio (567, 22.9%), platelet/lymphocyte ratio (572, 23.1%).

### 90-day mortality prediction model

**Fig. 1:**
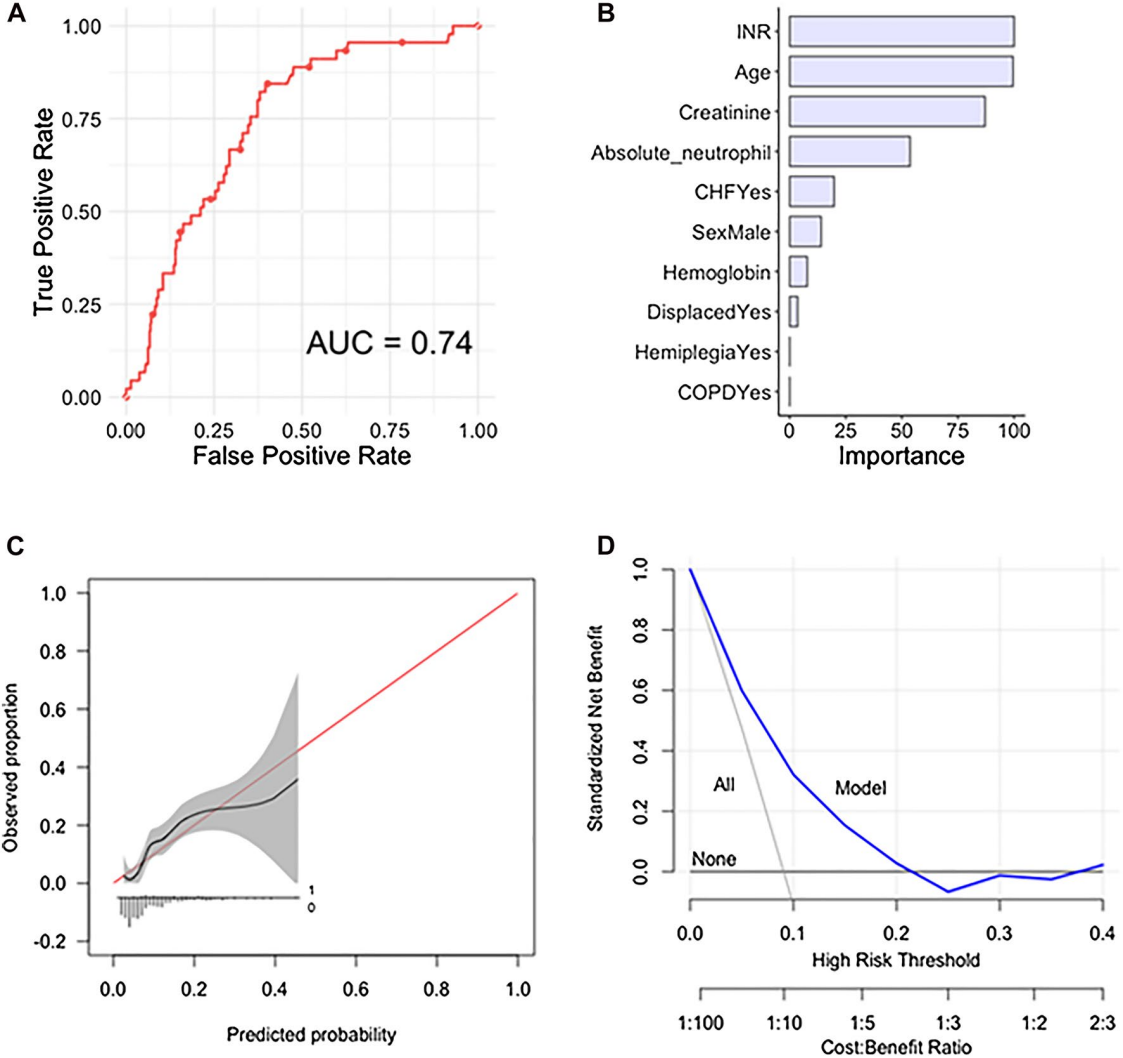
(**A**) Receiver operating curve, (**B**) global variable importance, (**C**) calibration plot and (**D**) decision curve analysis for the stochastic gradient boosting algorithm for prediction of 90 day mortality in the testing set, *n* = 495

The following variables were included after variable selection: (1) INR; (2) age; (3) creatinine level; (4) absolute neutrophil; (5) CHF; (6) male gender; (7) hemoglobin; (8) displaced fracture; (9) hemiplegia and (10) COPD (Fig. 1).

The performance of the conducted ML algorithms varied as measured by c-statistic from 0.53 to 0.74 in the independent testing set (Table 3) (performance of cross-validation on the training set can be found in Table 2). Model performance as assessed on calibration plot ranged from intercept − 0.08 to 0.15, and slope ranged from 0.71 to 2.13. The Brier scores ranged from 0.078 to 0.082 with Null model Brier score 0.83 (Table 3). The SGB algorithm was chosen as the final model with a c-statistic of 0.74, calibration intercept of − 0.05, calibration slope of 1.11 and a Brier score of 0.078.

**Table 2:** Algorithm performance on cross-validation of training set, *n* = 1983, mean (95% confidence interval)

	c-statistic	Calibration intercept	Calibration slope	Brier score
		Ninety Day Mortality		
Stochastic Gradient Boosting*	0.73 (0.71, 0.75)	0.20 (− 0.07, 0.47)	1.10 (0.98, 1.23)	0.077 (0.076, 0.079)
Random Forest	0.71 (0.70, 0.73)	− 0.59 (− 0.79, − 0.39)	0.65 (0.56, 0.74)	0.080 (0.079, 0.081)
Support Vector Machine	0.54 (0.51, 0.57)	− 1.06 (− 3.18, 1.06)	0.55 (− 0.37, 1.46)	0.083 (0.082, 0.083)
Neural Network	0.73 (0.71, 0.76)	0.02 (− 0.21, 0.25)	1.05 (0.93, 1.16)	0.078 (0.077, 0.079)
Elastic− Net Penalized Logistic Regression	0.74 (0.72, 0.76)	− 0.05 (− 0.29, 0.18)	0.98 (0.87, 1.09)	0.078 (0.076, 0.079)
		Two Year Mortality		
Stochastic Gradient Boosting	0.71 (0.70, 0.73)	− 0.04 (− 0.14, 0.06)	0.96 (0.88, 1.04)	0.16 (0.16, 0.17)
Random Forest	0.71 (0.69, 0.72)	0.00 (− 0.10, 0.10)	0.82 (0.75, 0.90)	0.16 (0.16, 0.17)
Support Vector Machine	0.64 (0.63, 0.66)	0.11 (− 0.11, 0.34)	1.09 (0.90, 1.28)	0.17 (0.17, 0.17)
Neural Network	0.71 (0.70, 0.73)	− 0.02 (− 0.12, 0.07)	0.99 (0.91, 1.08)	0.16 (0.16, 0.17)
Elastic− Net Penalized Logistic Regression*	0.72 (0.70, 0.73)	0.05 (− 0.07, 0.17)	1.05 (0.95, 1.16)	0.16 (0.16, 0.16)

NULL model Brier score: ninety day = 0.083, two year = 0.18

*AUC* area under the receiver operating curve

*Best-performing algorithm

**Table 3:** Algorithm performance in independent testing set, *n* = 495, mean (95% confidence interval)

	c-statistic	Calibration intercept	Calibration slope	Brier score
		Ninety Day Mortality		
Stochastic Gradient Boosting*	0.74 (0.67, 0.80)	− 0.05 (− 0.37, 0.26)	1.11 (0.73, 1.51)	0.078 (0.061, 0.098)
Random Forest	0.72 (0.64, 0.79)	0.15 (− 0.21, 0.45)	0.71 (0.38, 1.05)	0.082 (0.064, 0.103)
Support Vector Machine	0.53 (0.43, 0.60)	0.00 (− 0.30, 0.30)	2.13 (− 3.94, 8.21)	0.082 (0.063, 0.107)
Neural Network	0.71 (0.62, 0.78)	− 0.08 (− 0.40, 0.23)	0.94 (0.55, 1.34)	0.078 (0.060, 0.100)
Elastic− Net Penalized Logistic Regression	0.72 (0.63, 0.79)	− 0.01 (− 0.35, 0.28)	0.90 (0.53, 1.31)	0.078 (0.060, 0.098)
		Two Year Mortality		
Stochastic Gradient Boosting	0.69 (0.63, 0.74)	− 0.02 (− 0.24, 0.21)	0.90 (0.61, 1.19)	0.17 (0.15, 0.19)
Random Forest	0.70 (0.64, 0.75)	0.22 (− 0.03, 0.45)	0.83 (0.58, 1.12)	0.17 (0.14, 0.19)
Support Vector Machine	0.63 (0.57, 0.69)	0.01 (− 0.19, 0.24)	0.97 (0.50, 1.48)	0.17 (0.15, 0.19)
Neural Network	0.70 (0.64, 0.75)	− 0.04 (− 0.25, 0.18)	0.89 (0.60, 1.16)	0.16 (0.15, 0.18)
Elastic-Net Penalized Logistic Regression*	0.70 (0.63, 0.75)	− 0.03 (− 0.27, 0.19)	0.89 (0.62, 1.19)	0.16 (0.15, 0.18)

NULL model Brier score: Ninety Day = 0.083, Two Year = 0.18

AUC = area under the receiver operating curve;

*Best-performing algorithm

### 2-year mortality prediction model

**Fig. 2:**
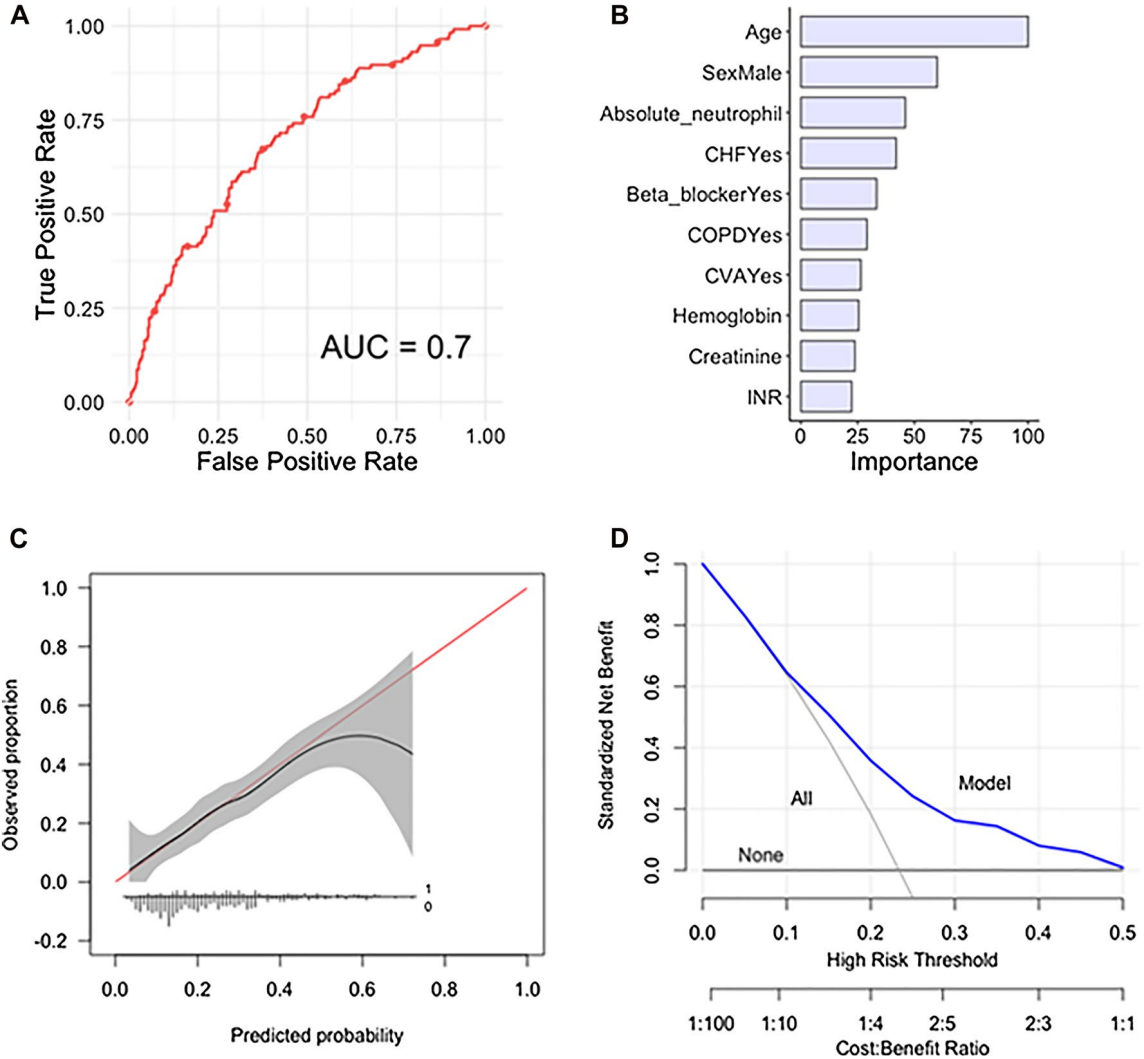
**A** Receiver operating curve, (**B**) global variable importance, (**C**) calibration plot and (**D**) decision curve analysis for the elastic-net penalized logistic regression algorithm for prediction of 2 year mortality in the testing set, *n* = 495

The following variables were included after variable selection: (1) age; (2) male gender; (3) absolute neutrophil; (4) CHF; (5) use of beta-blocker; (6) COPD; (7) CVA; (8) hemoglobin; (9) creatinine level and (10) INR (Fig. 2).

The performance of the conducted ML algorithms varied as measured by c-statistic from 0.63 to 0.70 in the independent testing set (Table 3) (performance of cross-validation on the training set can be found in Table 2). Model performance as assessed on calibration plot ranged from intercept − 0.04 to 0.22, and slope ranged from 0.83 to 0.97. The Brier scores ranged from 0.16 to 0.17 with Null model Brier score 0.18 (Table 3). The PLR algorithm was chosen as the final model with a c-statistic of 0.70, calibration intercept of -0.03, calibration slope of 0.89 and a Brier score of 0.16.

### Decision curve analysis

Decision curve analyses of both models revealed that decision changes based on the model outperformed as compared to the default strategies of changing management for all patients or for no patients (Figs. 1D and 2D). However, the clinical utility in relevant risk threshold ranges showed clearer benefit for the 2 year mortality model.

### Available web-application

The chosen algorithms were incorporated into a web-based application and deployed as open-access available tool for clinicians: https://sorg-apps.shinyapps.io/hipfracturemortality/.

### Individual patient-level explanation

**Fig. 3:**
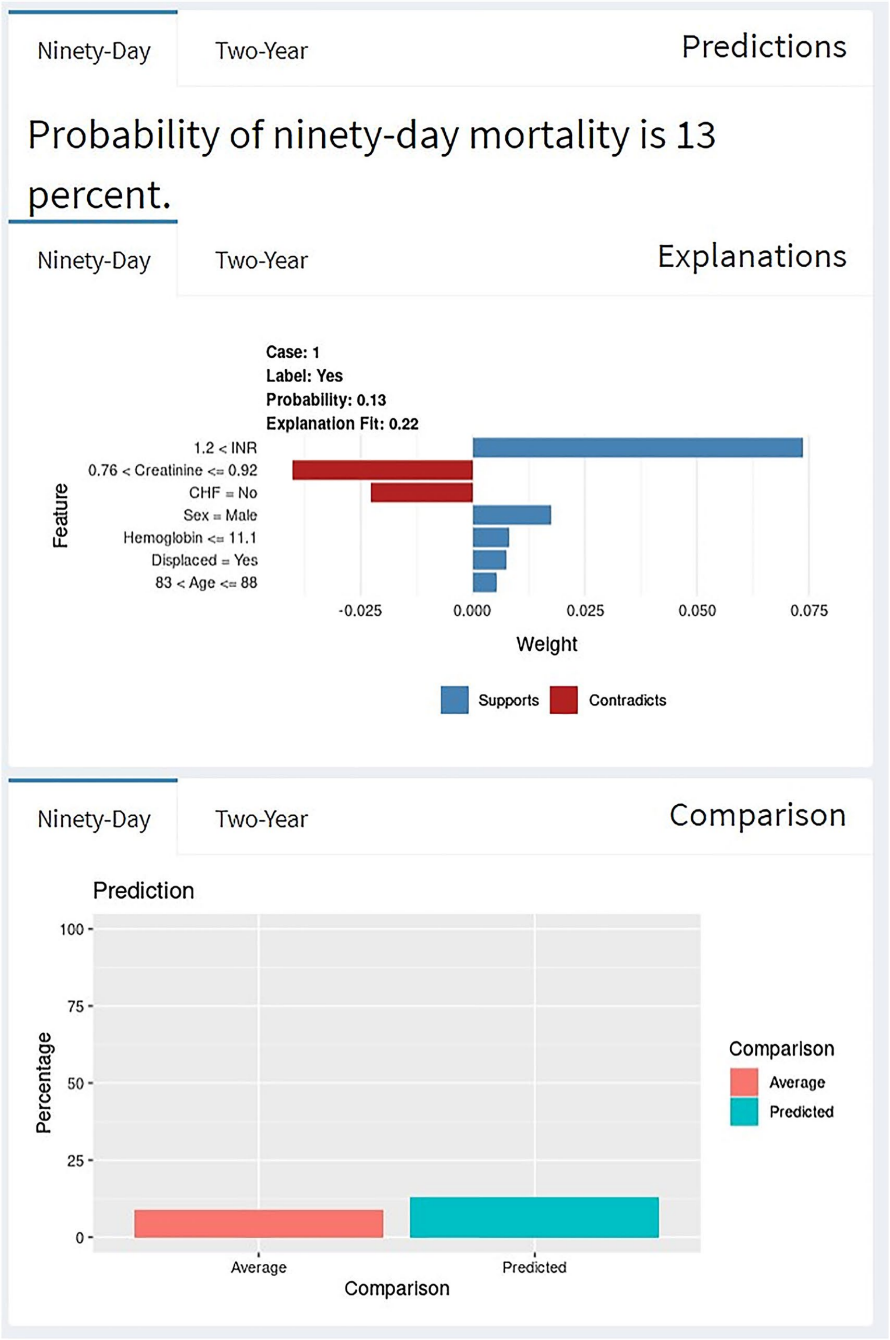
Example of individual patient-level explanation for 90 day mortality prediction

**Fig. 4:**
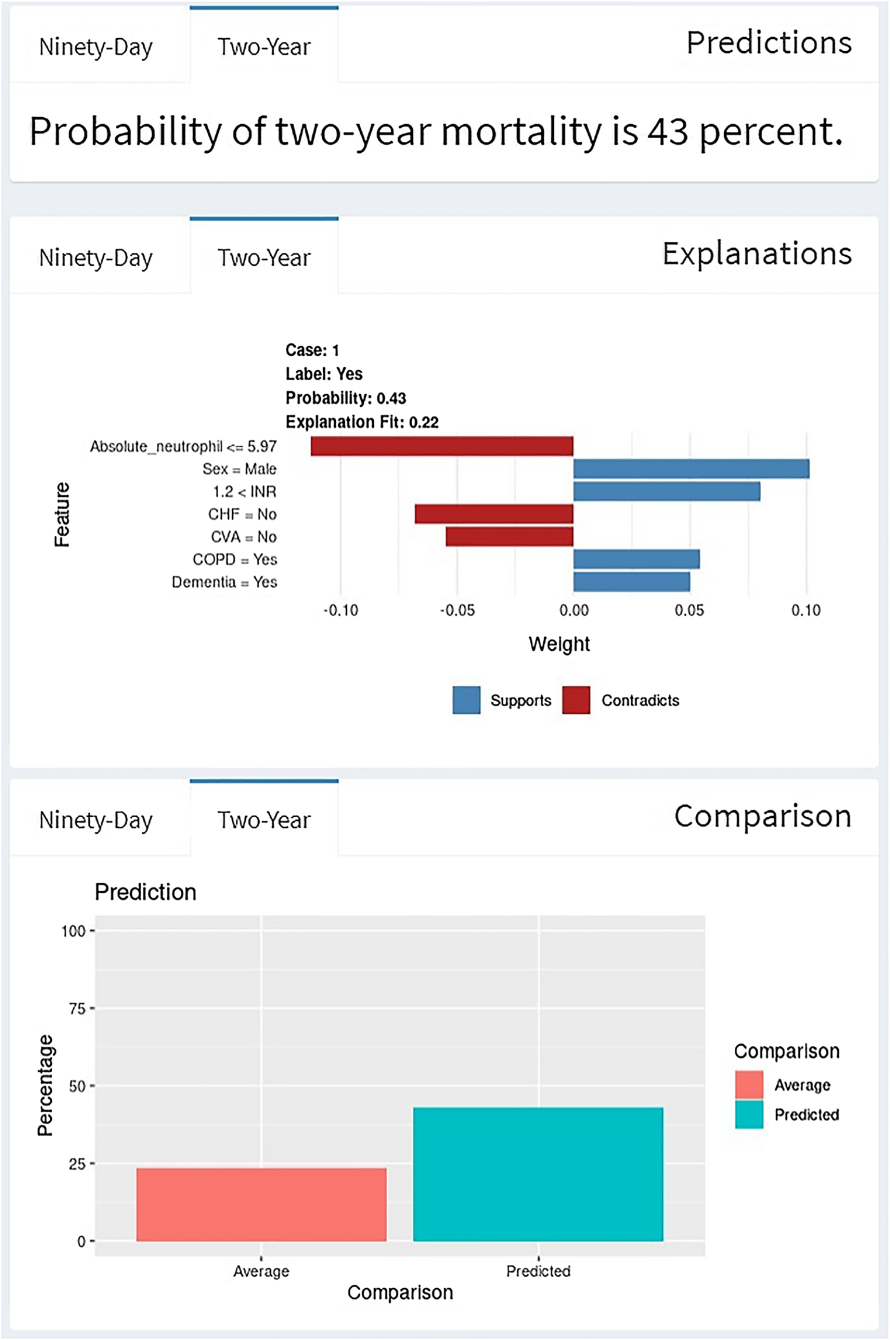
Example of individual patient-level explanation for 2 year mortality prediction

As an example, an 84 year-old male patient, after filling out the patient and injury characteristics values in the algorithm, this patient has a 13% and 43% chances of mortality in respectively 90 day and 2 year following femoral neck fracture surgery (Figs. 3 and 4).

Factors increasing the likelihood of 90 day mortality were an INR of 1.5, male gender, hemoglobin level of 9, sustaining a displaced fracture and an age of 84 years old. However, the lack of CHF and a creatinine level of 0.8 reduced the likelihood of mortality following femoral neck fracture surgery. The predicted probability (13%) was higher than the average probability in the total patient cohort (9.1%) (Fig. 3).

Factors increasing the likelihood of 2 year mortality were male gender, a history of COPD and dementia. However, a low absolute neutrophil level of 0.8 and the lack of CHF or having a history of CVA reduced the likelihood of mortality. The predicted probability (43%) was higher than the average probability (23.5%) (Fig. 4).

## Discussion

The aim of this study was to develop and internally validate a clinical prediction model that can predict 90 day and 2 year mortality in femoral neck fracture patients aged 65 years or above to aid the challenging treatment decision-making. The developed and internally validated models show promise in estimating mortality in this frail patient population.

### Limitations

The results of this study should be viewed in light of several limitations. First, the study was a retrospective study beholden to limitations inherent to such research design and prospective validation remains to be evaluated. Second, the mortality rate in our cohort was relatively low compared to other populations of hip fracture patients [38]. This resulted in predicted probabilities as shown in the calibration plots, up to 50% and 80% risk for respectively 90 day and 2 year mortality. This means that our model is likely more accurate in healthier hip fracture patients. To ensure external validation, our model should be validated in a cohort with representative rates, and future studies should assess the transportability of the developed algorithm to datasets with patients with higher mortality rates. Third, for this study, we chose a 80/20 ratio for data splitting into training and test set, which has been mostly used in previous literature [20–22, 39]. There is no fixed rule for the ratio of data splitting but a different ratio for algorithm training may have led to different model performances. Fourth, preoperative risk stratification for mortality is needed to guide the difficult treatment decision-making, although intraoperative and postoperative factors associated with complications, such as reoperation or postoperative infection, may be confounding with mortality after surgery. Future research may estimate this influence looking at causality for confounding factors [40]. Fifth, patients were included in the study undergoing femoral neck fracture surgery. However, patients who were suspected by the clinician of a very short survival prediction (e.g. 30 day) were chosen to be treated conservatively and were not investigated in this study. In future studies, both conservative and surgical treated patients should be included to optimize mortality prediction in all patients sustaining a femoral neck fracture to guide the challenging treatment decision-making (i.e. whether to operate or not?). Sixth, evaluating possible co-injuries occurring during trauma, some of which may cause significant disability, may influence survival outcome. Evaluating these co-injuries and calculating their injury severity score may have had an influence as candidate input variable on the model performance. In addition, we did not investigate the influence of the presence of advanced directives, which may influence the decision-making process in patients aged 65 years or above. In future research, when comparing treatment effects in conservatively and operatively treated patients, we recommend these influences to be investigated. Lastly, the 2 year mortality was chosen on the basis of endpoints in prior prospective randomized controlled trials [5, 6]. The 90 days was chosen to predict short-term mortality and accounts for a possible underestimation in outcomes seen with only a 30 day mortality. From a patient and provider perspective, a death 90 days post hip fracture is just as significant as one within 30 days. It takes in to account not just acute in-hospital complications but also short-term complications that may occur in skilled nursing facility and discharge to the community. There is growing evidence in other specialties that 30-day mortality underestimates short-term mortality [41, 42]. Future studies may additionally investigate earlier time points, such as 30 days or 1 year.

### Findings

In the ranges of risk where we think clinical utility of the model is to be expected, the 2 year model clearly adds clinical utility over treating everyone or none with total hip arthroplasty. However, we assumed a more simplified scenario, since there are multiple treatment options available, namely nonoperative management, surgical fixation and arthroplasty surgery. The 90 day mortality model might add clinical utility for decisions between these tiered treatment options, which are more subtle and complex to assume. Moreover, clinical utility should be reassessed after external validation, and with input from multiple institutions from different countries. If found to be externally valid (generalizable to independent populations), future studies should prospectively evaluate the developed and validated tool. In patients with limited life expectancy, patients predicted with a high risk of short-term mortality, nonoperative management might be a viable option in the shared decision-making process compared to surgical fixation [8]. If patients have a high chance of surviving beyond the 90 day endpoint, surgical management would be in place [43]. Frail patients with a nondisplaced hip fracture may be favored to surgical fixation compared to arthroplasty surgery [6, 18]. However, arthroplasty is associated with a lower risk of reoperation and better long-term functional outcomes, at the cost of greater infection rates, blood loss, and operative time and possibly an increase in early mortality rates and may be recommended in patients with a longer-term life expectancy (e.g., high probability of surviving beyond the 2 year endpoint) [44].

When aiming to develop a prediction model that is applicable in daily practice, variables should be included in the trained algorithm that are readily available and use of definitions that are in line with daily practice should be followed. In this study, variables derived from variable selection are clinically readily available and in line with daily practice. It is important to emphasize that treatment decision-making should not be solely based on the outcome of an individualized probability calculator. The orthopaedic surgeon should discuss the available treatment options and reach a treatment decision following a shared decision-making process. Prediction of mortality is only one of the aspects to be considered in treatment decision-making.

The most important factors associated with a greater risk of 90 day mortality included in the SGB algorithm were INR, age, creatinine level, absolute neutrophil, CHF, male gender, hemoglobin level, displaced fracture, hemiplegia and COPD. For 2 year mortality, the most important factors were age, male gender, absolute neutrophil, CHF, use of beta-blocker, COPD, CVA, hemoglobin, creatinine level and INR. Our findings are in line with previous research on proximal femoral neck fractures in general and broader populations. Regarding age and sex, prior studies revealed a higher risk for higher age and the male gender [45–47]. The effect of CHF, CVA and COPD is in line with the high risk reported for a higher ASA classification in earlier studies [48, 49]. A possible explanation for this effect might be a lower physical condition of the patient at baseline and therefore a less adequate recovery after complications (e.g. pneumonia). Another explanation for comorbidities in general could be a lower life expectancy as a result of the comorbidity itself. In regard to displacement of the fracture, a reasonable explanation for the higher risk might be the disruption of the vascularization of the femoral head and the tendency that a displaced fracture comes from a frailer patient to start with where more displacement occurred compared to a younger patient (with the same level energy of trauma). This could lead to multiple complications and secondary surgery eventually resulting in death [50]. The prognostic value of laboratory characteristics in predicting mortality after hip surgery is a less explored subject. But the elevation of creatinine and absolute neutrophil count reflects respectively declined renal function and inflammation [51]. Which again is linked to a higher ASA score and a lower baseline physical condition. Whereas a higher INR is reflecting the inability to coagulate and most likely the use of anticoagulants, resulting in a higher risk for bleeding and as a result of this a higher risk for morbidity and mortality [46, 51]. On the contrary a lower hemoglobin is related to chronic comorbidities, which might reflect in a lower odds for mortality for higher hemoglobin levels [51].

Over the recent years, a lot of research has been done predicting mortality in femoral neck fracture patients. The greater part of these tools developed made an estimation of risk based on age, gender and in general the presence of comorbidity [52, 53], whereas the other part looked at postoperative factors, such as early ambulation after surgery and postoperative lab values [54, 55]. In contrast to the broader presence of comorbidity, our study used the ability of ML algorithms to differ between the effects of different types of comorbidity in a large database to estimate the individual value of each factor. This resulted in a more patient centered prediction tool.

### Future perspectives

External validation is essential before testing and implementing the ML algorithm in clinical practice. Subsequently, a prospective observational study of the comparison of the current ML model prediction compared to a physician’s prediction of mortality can assess the clinical usefulness of the developed model. This will assess if the model’s prediction was more accurate than those of the treating physician [56]. An internally and externally validated algorithms can then be integrated into the electronic health record with an active feedback loop to improve the model performance and ultimately be integrated in the clinical workflow [57, 58].

### Conclusion

In summary, the developed and internally validated clinical prediction model effectively predicts 90 day and 2 year mortality in femoral neck fracture patients aged 65 years or above with good model performance on discrimination, calibration and Brier score. Especially the model for 2 year mortality would likely improve the challenging treatment decision-making. Nevertheless, the model first requires external validation in an independent cohort. The model can be freely accessed: https://sorg-apps.shinyapps.io/hipfracturemortality/.

## Supplementary Information

Below is the link to the electronic supplementary material.

Supplementary file1 TRIPOD checklist: prediction model validation (DOCX 88 kb)

## References

[1] Topol EJ (2019). High-performance medicine: the convergence of human and artificial intelligence. Nat Med.

[2] Panch T, Szolovits P, Atun R (2018). Artificial intelligence, machine learning and health systems. J Glob Health.

[3] Fontana MA, Lyman S, Sarker GK, Padgett DE, MacLean CH (2019). Can machine learning algorithms predict which patients will achieve minimally clinically important differences from total joint arthroplasty?. Clin Orthop Relat Res.

[4] Tran B, Vu G, Ha G, Vuong Q-H, Ho M-T, Vuong T-T (2019). Global evolution of research in artificial intelligence in health and medicine: a bibliometric study. J Clin Med.

[5] Viveiros H, Lieshout EMM Van, Nutsohra S, Gmbh S, Ingelheim B, Diagnostics R, et al. Fracture fixation in the operative management of hip fractures (FAITH): an international, multicentre, randomised controlled trial. 2017;389:1519–27. 10.1016/S0140-6736(17)30066-1 PMC5597430 28262269

[6] HEALTH Investigators, Bhandari M, Einhorn T, et al. Total Hip Arthroplasty or Hemiarthroplasty for Hip Fracture. N Engl J Med. 2019;381:2199–208. 10.1056/NEJMoa1906190 31557429

[7] Joosse P, Loggers SAI, Van de Ree CLPM, Van Balen R, Steens J, Zuurmond RG (2019). The value of nonoperative versus operative treatment of frail institutionalized elderly patients with a proximal femoral fracture in the shade of life (FRAIL-HIP); protocol for a multicenter observational cohort study. BMC Geriatr.

[8] Loggers SAI, Willems HC, Van Balen R, Gosens T, Polinder S, Ponsen KJ (2022). Evaluation of quality of life after nonoperative or operative management of proximal femoral fractures in frail institutionalized patients: the FRAIL-HIP study. JAMA Surg.

[9] Keating JF, Grant A, Masson M, Scott NW, Forbes JF. Randomized comparison of reduction and fixation, bipolar hemiarthroplasty, and total hip arthroplasty. Treatment of displaced intracapsular hip fractures in healthy older patients. J Bone Joint Surg Am; 2006;88:249–60. 10.2106/JBJS.E.00215 16452734

[10] Rogmark C, Carlsson A, Johnell O, Sernbo I. A prospective randomised trial of internal fixation versus arthroplasty for displaced fractures of the neck of the femur. Functional outcome for 450 patients at two years. J Bone Joint Surg Br.; 2002;84:183–8. 10.1302/0301-620x.84b2.11923 11922358

[11] Schuijt HJ, Bos J, Smeeing DPJ, Geraghty O, van der Velde D (2021). Predictors of 30-day mortality in orthogeriatric fracture patients aged 85 years or above admitted from the emergency department. Eur J trauma Emerg Surg Off Publ Eur Trauma Soc.

[12] Chow J, Kuza CM (2022). Predicting mortality in elderly trauma patients: a review of the current literature. Curr Opin Anaesthesiol.

[13] Beigmohammadi MT, Amoozadeh L, Rezaei Motlagh F, Rahimi M, Maghsoudloo M, Jafarnejad B (2022). Mortality predictive value of APACHE II and SOFA scores in COVID-19 patients in the intensive care unit. Can Respir J.

[14] Wiles MD, Moran CG, Sahota O, Moppett IK (2011). Nottingham Hip Fracture Score as a predictor of one year mortality in patients undergoing surgical repair of fractured neck of femur. Br J Anaesth.

[15] Schuijt HJ, Smeeing DPJ, Würdemann FS, Hegeman JH, Geraghty OC, Houwert RM (2020). Development and Internal Validation of a Prediction Model for In-Hospital Mortality in Geriatric Patients With a Hip Fracture. J Orthop Trauma..

[16] DeBaun MR, Chavez G, Fithian A, Oladeji K, Van Rysselberghe N, Goodnough LH (2021). Artificial neural networks predict 30-day mortality after hip fracture: insights from machine learning. J Am Acad Orthop Surg.

[17] Werner M, Macke C, Gogol M, Krettek C, Liodakis E (2021). Differences in hip fracture care in Europe: a systematic review of recent annual reports of hip fracture registries. Eur J Trauma Emerg Surg..

[18] Fixation using Alternative Implants for the Treatment of Hip fractures (FAITH) Investigators. Fracture fixation in the operative management of hip fractures (FAITH): an international, multicentre, randomised controlled trial. Lancet. 2017;389:1519–1527. Available from: https://europepmc.org/articles/PMC5597430 10.1016/S0140-6736(17)30066-1 PMC5597430 28262269

[19] Oosterhoff JHF, Karhade AV, Oberai T, Franco-Garcia E, Doornberg JN, Schwab JH (2021). Prediction of postoperative delirium in geriatric hip fracture patients: a clinical prediction model using machine learning algorithms. Geriatr Orthop Surg Rehabil.

[20] Karhade AV, Thio QCBS, Ogink PT, Shah AA, Bono CM, Oh KS (2019). Development of machine learning algorithms for prediction of 30-day mortality after surgery for spinal metastasis. Clin Neurosurg.

[21] Shah AA, Karhade AV, Bono CM, Harris MB, Nelson SB, Schwab JH (2019). Development of a machine learning algorithm for prediction of failure of nonoperative management in spinal epidural abscess. Spine J.

[22] Bongers MER, Thio QCBS, Karhade AV, Stor ML, Raskin KA, Lozano Calderon SA (2019). Does the SORG algorithm predict 5-year survival in patients with chondrosarcoma? An external validation. Clin Orthop Relat Res.

[23] Meinberg EG, Agel J, Roberts CS, Karam MD, Kellam JF. Fracture and Dislocation Classification Compendium-2018. J Orthop Trauma.; 2018;32 Suppl 1:S1–170. 10.1097/BOT.0000000000001063 29256945

[24] Garland A, Bülow E, Lenguerrand E, Blom A, Wilkinson M, Sayers A, et al. Prediction of 90-day mortality after total hip arthroplasty. Bone Joint J.; 2021;103-B:469–78. 10.1302/0301-620X.103B3.BJJ-2020-1249.R1 33641419

[25] Stekhoven DJ, Buhlmann P (2012). MissForest–non-parametric missing value imputation for mixed-type data. Bioinformatics.

[26] Strobl C, Boulesteix A-L, Kneib T, Augustin T, Zeileis A (2008). Conditional variable importance for random forests. BMC Bioinform.

[27] Riley RD, Ensor J, Snell KIE, Harrell FE, Martin GP, Reitsma JB, et al. Calculating the sample size required for developing a clinical prediction model. BMJ Publishing Group Ltd. 2020;18(368) 10.1136/bmj.m441 32188600

[28] Karhade AV, Ogink PT, Thio QCBS, Cha TD, Gormley WB, Hershman SH (2019). Development of machine learning algorithms for prediction of prolonged opioid prescription after surgery for lumbar disc herniation. Spine J.

[29] Karhade A V., Ogink PT, Thio QCBS, Cha TD, Hershman SH, Schoenfeld AJ, et al. Discharge Disposition After Anterior Cervical Discectomy and Fusion. World Neurosurg. Elsevier Inc.; 2019;132:e14–20. 10.1016/j.wneu.2019.09.026 31521753

[30] Steyerberg EW, Vickers AJ, Cook NR, Gerds T, Gonen M, Obuchowski N (2010). Assessing the performance of prediction models: a framework for traditional and novel measures. Epidemiology.

[31] Cox DR (1958). Two Further Applications of a Model for Binary Regression. Biometrika.

[32] Steyerberg EW, Vergouwe Y (2014). Towards better clinical prediction models: seven steps for development and an ABCD for validation. Eur Heart J.

[33] VanCalster B, Vickers AJ (2015). Calibration of risk prediction models: impact on decision-analytic performance. Med Decis Making.

[34] Vickers AJ, Elkin EB (2006). Decision curve analysis: a novel method for evaluating prediction models. Med Decis Making.

[35] Vickers AJ, van Calster B, Steyerberg EW (2019). A simple, step-by-step guide to interpreting decision curve analysis. Diagnostic Progn Res.

[36] Dhinakaran A. A Look Into Global, Cohort and Local Model Explainability [Internet]. Available from: https://towardsdatascience.com/a-look-into-global-cohort-and-local-model-explainability-973bd449969f. Accessed 15 Nov 2021.

[37] Collins GS, Reitsma JB, Altman DG, Moons KGM (2015). Transparent reporting of a multivariable prediction model for individual prognosis or diagnosis (TRIPOD): the TRIPOD statement. BMJ.

[38] Xu BY, Yan S, Low LL, Vasanwala FF, Low SG (2019). Predictors of poor functional outcomes and mortality in patients with hip fracture: a systematic review. BMC Musculoskelet Disord.

[39] Ogink PT, Karhade AV, Thio QCBS, Gormley WB, Oner FC, Verlaan JJ (2019). Predicting discharge placement after elective surgery for lumbar spinal stenosis using machine learning methods. Eur spine J Off Publ Eur Spine Soc Eur Spinal Deform Soc Eur Sect Cerv Spine Res Soc.

[40] Badgeley MA, Zech JR, Oakden-Rayner L, Glicksberg BS, Liu M, Gale W (2019). Deep learning predicts hip fracture using confounding patient and healthcare variables. npj Digit Med.

[41] Hirji S, McGurk S, Kiehm S, Ejiofor J, Ramirez-Del Val F, Kolkailah AA (2020). Utility of 90-day mortality vs 30-day mortality as a quality metric for transcatheter and surgical aortic valve replacement outcomes. JAMA Cardiol.

[42] Visser BC, Keegan H, Martin M, Wren SM (2009). Death after colectomy: it’s later than we think. Arch Surg.

[43] Bhandari M, Swiontkowski M (2017). Management of acute hip fracture. N Engl J Med.

[44] Bhandari M, Devereaux PJ, Swiontkowski MF, Tornetta P 3rd, Obremskey W, Koval KJ, et al. Internal fixation compared with arthroplasty for displaced fractures of the femoral neck. A meta-analysis. J Bone Joint Surg Am.; 2003;85:1673–81. 10.2106/00004623-200309000-00004 12954824

[45] Vosoughi AR, Emami MJ, Pourabbas B, Mahdaviazad H (2017). Factors increasing mortality of the elderly following hip fracture surgery: role of body mass index, age, and smoking. Musculoskelet Surg.

[46] Rosso F, Dettoni F, Bonasia DE, Olivero F, Mattei L, Bruzzone M, et al. Prognostic factors for mortality after hip fracture: Operation within 48 hours is mandatory. Injury.; 2016;47 Suppl 4:S91–7. 10.1016/j.injury.2016.07.055 27546722

[47] Karademir G, Bilgin Y, Erşen A, Polat G, Buget MI, Demirel M (2015). Hip fractures in patients older than 75 years old: Retrospective analysis for prognostic factors. Int J Surg.

[48] Bilsel K, Erdil M, Gulabi D, Elmadag M, Cengiz O, Sen C (2013). Factors affecting mortality after hip fracture surgery: a retrospective analysis of 578 patients. Eur J Orthop Surg Traumatol.

[49] Paksima N, Koval KJ, Aharanoff G, Walsh M, Kubiak EN, Zuckerman JD (2008). Predictors of mortality after hip fracture: a 10-year prospective study. Bull NYU Hosp Jt Dis.

[50] Swiontkowski MF (1994). Current concepts review: Intracapsular fractures of the hip. J Bone Jt Surg - Ser A.

[51] Kumar P, Clark M. Kumar and Clark’s Clinical Medicine. 9th ed. Elsevier Ltd; 2017.

[52] Morri M, Ambrosi E, Chiari P, Orlandi Magli A, Gazineo D, D’ Alessandro F, et al. One-year mortality after hip fracture surgery and prognostic factors: a prospective cohort study. Sci Rep. 2019;9:18718. 10.1038/s41598-019-55196-6 10.1038/s41598-019-55196-6 PMC6904473 31822743

[53] Tsang C, Boulton C, Burgon V, Johansen A, Wakeman R, Cromwell DA (2017). Predicting 30-day mortality after hip fracture surgery. Bone Jt Res.

[54] Heiden JJ, Goodin SR, Mormino MA, Siebler JC, Putnam SM, Lyden ER (2021). Early ambulation after hip fracture surgery is associated with decreased 30-day mortality. J Am Acad Orthop Surg.

[55] Blanco JF, da Casa C, Pablos-Hernández C, González-Ramírez A, Julián-Enríquez JM, Díaz-Álvarez A (2021). 30-day mortality after hip fracture surgery: influence of postoperative factors. PLoS One.

[56] Gensheimer MF, Aggarwal S, Benson KRK, Carter JN, Henry AS, Wood DJ (2020). Automated model versus treating physician for predicting survival time of patients with metastatic cancer. J Am Med Inform Assoc.

[57] Oosterhoff J, Doornberg J. Artificial Intelligence in Orthopaedics: False Hope or Not? A Narrative Review along the line of Gartner’s Hype Cycle. EFORT Open Rev. 2020;5. 10.1302/2058-5241.5.190092 PMC7608572 33204501

[58] Oosterhoff J, Thio Q, Groot O, Bongers M, Ghaednia H, Karhade A, et al. Integration of automated predictive analytics into electronic health records: can spine surgery applications lead the way using SMART on FHIR and CDS Hooks? Semin Spine Surg. 2021;33(2).

